# Developing a Support Program for Adult Children of Parents with Mental Illness: A Delphi Study

**DOI:** 10.1007/s10597-022-00999-7

**Published:** 2022-07-01

**Authors:** Pamela M. Patrick, Andrea E. Reupert, Louise A. McLean, Emily Berger

**Affiliations:** Faculty of Education, School of Educational Psychology & Counselling, Monash University, 19 Ancora Imparo Way, Clayton, VIC 3800 Australia

**Keywords:** Adult children, Parents with mental illness, Program development, Support services, Help-seeking, Delphi technique

## Abstract

Parental mental illness can have long-lasting impacts on a child’s life. Although programs exist in supporting the needs of young children, there remains a paucity in programs that address the needs of adult children. A two-round Delphi study with adult children, academics and clinicians who have experience with parental mental illness was employed. A total of 45 and 24 participants participated in rounds one and two respectively. Open-ended questions in round one around program design and content were thematically analysed, and subsequently rated in round two. Adult children specifically identified four topics of need: (i) managing multiple roles, (ii) emotional regulation, (iii) setting relational boundaries and (iv) transition to parenthood. Current results provide the foundation for the development of modular programs that could be pilot tested with adult children who grew up with parents with mental illness.

## Introduction

Parental mental illness (PMI) can pose a myriad of challenges for children and parents alike (Afzelius et al., 2018). In Australia, an estimated 23.3% of children currently reside in households where at least one parent has a mental illness (Maybery et al., 2009). Over the last two decades, research efforts have led to the development of parent, child and family-centred interventions aimed to prevent the transmission of mental illness to children in families where a parent has a mental illness (Siegenthaler et al., 2012). At the same time there is a lack of interventions for adult children who grew up in these families.

There are several interventions available to children whose parents have a mental illness that focus on promoting psychoeducation and adaptive coping, within a peer group setting. Given young people’s preference for online support (Matar, 2018), there are now several online interventions, including ‘Kopstoring’ and ‘Survivalkid’, both developed in the Netherlands, which offer a platform for children to discuss their experiences living with PMI in conjunction with peers and health care professionals (Drost et al., 2011). Other online interventions have also been developed in Australia (mi.spot; Reupert et al., 2019) and Norway (Trondsen & Tjora, 2014). At present, the evidence-base around the efficacies of these interventions remain emergent and have age limits for participation (e.g., between 18 and 25 years) (Matar et al., 2018; Reupert et al., 2013). Hence, there is a growing need to develop similar interventions that address the unique circumstances and needs of adult children who grew up with parents with a mental illness.

Adult children are defined as the middle generation within a family unit, who sometimes assume dual caretaking responsibilities for their parent who has a mental illness (first generation) and their dependent children (third generation). Several themes have emerged in the small, but growing number of research studies focusing on adult children. Adult children highlight issues around parentification and burnout resulting from increased caregiving responsibilities (Knutsson-Medin et al., 2007). Feelings of abandonment, loneliness, difficulties forming trust in adulthood, as well as anger or envy of peers are some additional themes reported in the literature to date (Knutsson-Medin et al., 2007; O’Connell, 2008). Often issues that arise in childhood can have a long-lasting and profound impact on other areas of one’s life years later, including their own parenting style (Patrick et al., 2019). This underscores the need to ensure there are adequate supports available to adult children, who grew up with a parent with mental illness. Furthermore, adult children of parents with mental illness grew up in a time where awareness and support groups for families with parental mental illness were scarce. When little is done to address problematic experiences in childhood, new challenges may emerge as these individuals’ transition into adulthood. Hence, it is vital to gather multi stakeholder perspectives on intervention needs for adult children of parents with a mental illness.

## Method

A two-round Delphi study was conducted using online questionnaires. Utilising a systematic process of sequential questionnaires, the Delphi technique allows for aggregated feedback to be obtained from a group of experts in a specific area of inquiry (Goodman, 1987; Powell, 2003). The Delphi method minimises domination by powerful individuals in a group by drawing on anonymised data and can contribute to the expression of novel ideas and accurate feedback (Hasson et al., 2000).

### Recruitment and Inclusion Criteria

Experts were classified as those with lived experience (i.e., adult children), individuals with research (i.e., researchers/ academics) and clinical (i.e., clinicians) experience working with families where a parent has a mental illness. Although evidence points to the importance of including ‘consumer/survivor engagement’ to enhance policy development and research, such practices remain tokenistic (Daya et al., 2020; Read & Maslin-Prothero, 2011). The Delphi study provided adult children with a safe space to communicate their views. The decision was also made to include clinicians and researchers, as these groups are likely to be program facilitators and able to provide current implementation perspectives on program delivery. Purposive sampling was carried out by contacting adult children who had taken part in an earlier study as part of a larger PhD project. These individuals were requested to inform their personal networks (e.g., siblings and friends) about the study. The research team’s professional networks and social media were utilised to recruit academics and clinicians.

### Participants in Round One

A total of 45 participants took part in round one. Participants included 16 adult children with lived experience (aged 20–67 years; *M* = 39.13, *SD* = 12.71), 12 clinicians (aged 36–71 years;* M* = 51.3, *SD* = 10.3), and 17 academics (aged 29–71 years; *M* = 50.3, *SD* = 13.5). The professional experience of clinicians and academics was 5–30 years and 2–34 years respectively. Additional demographic information for each subgroup is provided in Table 1.

**Table 1:** Demographic data of participants from round one

	Adult Children (*n* = 16)	Clinician (*n* = 12)	Academics (*n* = 17)
Gender			
Female	13	10	11
Male	3	2	6
Nationality			
Australian	10	9	4
British	–	1	2
Chinese	–	–	1
Caucasian/Asian	1	–	–
Dutch	1	1	4
Finnish	2	–	–
Irish	1	1	4
Norwegian	1	–	1
Marital status			
Single	4	–	–
Married	6	–	–
De facto	2	–	–
Divorced/Separated	4	–	–
Employment status			
Full-time	3	–	–
Part-time	8	–	–
Unemployed, seeking work	2	–	–
Others: completing studies	3	–	–
Personal history with mental illness			
Ongoing	2	–	–
In the past	7	–	–
No history with mental illness	7	–	–
Participants who has/had a parent with mental illness	16	1	5
Nature of parent’s mental illness^a^			
Schizophrenia spectrum and other psychotic disorders	6	–	–
Bipolar and related disorders	7	–	1
Depressive disorders	7	–	2
Anxiety disorders	–	–	1
Obsessive–compulsive and related disorders	–	–	–
Trauma and stress related disorders	2	–	–
Substance-related and addictive disorders	1	–	2
Not formally diagnosed	–	–	1
Occupation			
Mental Health Nurse	–	1	1
Psychiatrist	–	1	2
Psychologist	–	1	10
Social Worker	–	8	2
Occupational Therapist	–	1	1
Unspecified	–	–	1

^a^Numbers are not cumulative due to co-morbid or multiple diagnoses

### Participants in Round Two

Round two of the Delphi study consisted of 24 participants, with 11 of these individuals being repeat participants who provided data in round one. Amongst adult children, clinicians and academics, there were four, two and five repeat participants respectively (i.e., provided responses in rounds 1 and 2). Nine adult children with lived experience between ages 25–49 (*M* = 39.2, *SD* = 8.03) were involved. Along with seven academics aged between 33–67 years (*M* = 49.7, *SD* = 12.4) and eight clinicians aged 28–60 (*M* = 48.8, *SD* = 9.1). The professional experience of clinicians and academics was 5–30 years within each group. Additional demographic information for each subgroup is provided in Table 2.

**Table 2:** Demographic data of participants from round two

	Adult Children (*n* = 9)	Clinician (*n* = 8)	Academics (*n* = 7)
Gender			
Female	8		4
Male	1		3
Nationality			
Australian	7		1
British			1
Chinese			–
Caucasian/Asian			–
Dutch	1		2
Finnish			–
Greek			1
Irish	1		1
Norwegian			1
Marital status			
Single	1	–	–
Married	4	–	–
De facto	3	–	–
Divorced/Separated	1	–	–
Employment status			
Full-time	2	–	–
Part-time	6	–	–
Unemployed, not seeking work	1	–	–
Others: completing studies	–	–	–
Personal history with mental illness			
Ongoing	0	–	–
In the past	4	–	–
No history with mental illness	5	–	–
Participants who has/had a parent with mental illness	9	2	4
Nature of parent’s mental illness^a^			
Schizophrenia spectrum and other psychotic disorders	4	–	–
Bipolar and related disorders	3	–	1
Depressive disorders	4	1	1
Anxiety disorders	2	1	1
Obsessive–compulsive and related disorders		–	–
Trauma and stress related disorders	3	–	–
Substance-related and addictive disorders	1	–	–
Not formally diagnosed	0		2
Occupation^b^			
Mental Health Nurse	–	3	1
Psychologist	–	2	4
Social Worker	–	4	1
Teacher	–	–	1

^a^Numbers are not cumulative due to co-morbid or multiple diagnoses

^b^Numbers are not cumulative due to some participants having more than one occupation

### Questionnaire

The questionnaire in round one consisted of nine questions, with a combination of open-ended and polar questions. An example of an open-ended question was: ‘*based on your experience, what are some challenges that adult children of parents with mental illness are likely to experience, if any?*’ Polar questions that required a ‘yes’ or ‘no’ response were followed by a sub-question that required participants to provide further elaboration for their chosen response. An example of a polar question: ‘*do you think there is a need for an online forum for adult children of parents with mental illness? Please specify your reason for your response above*’. In round one, open-ended questions are useful as it enables the generation of a wide range of responses from participants.

Round one responses were thematically analysed (Braun & Clarke, 2006). These themes were then used as options for rank and rating questions in round two. The questions required participants to either rate the level of importance on a 5-point Likert scale from 1 (not at all important) to 5 (absolutely important) or rank items based on their order of importance. For example, *‘please rank the most appropriate number of sessions for a program targeting adult children’*, from 1 ‘most appropriate’ to 4 ‘least appropriate’. Descriptive analysis, frequencies and mean ranks in round two were computed using IBM SPSS Statistics Version 20.

### Establishing Consensus for Rating Questions

Others have argued for consensus to be based on percentiles of agreement, as well as through pre-established means or medians or interquartile ranges (Hallowell & Gambatese, 2010; Hasson et al., 2000). Accordingly, mean and standard deviation thresholds were chosen to mirror previous Delphi studies in mental health and counselling (Neuer Colburn et al., 2016; Runyan et al., 2018). Out of a 5-point likert scale, a mean threshold of 4.0 or higher was set for inclusion. A threshold of 4.0 or higher was indicative of at least 80% agreement amongst panel members, which signified a relatively high level of consensus. A standard deviation threshold of 0.85 or less was established to indicate a reasonable degree of consensus in the range of responses to each item (Neuer Colburn et al., 2016; Runyan et al., 2018). Any item after round two that had a mean value of 4.0 or higher and a standard deviation of 0.85 or less was defined as having obtained consensus among expert panellists.

### Establishing Consensus for Ranking Questions

An option that received a rank of ‘1’ was most important. To derive aggregated scores across each expert group, each rank was awarded a score: rank 1 = 3 points, rank 2 = 2 points and rank 3 = 1 point (Stoner et al., 2017). This method of calculation awarded highest points to the option ranked first and the lowest point to the option ranked third by each panel member. The option with the highest aggregated point signified the most preferred option.

## Results: Round One

### Challenges Experienced by Adult Children

Panellists were invited to identify common challenges experienced by adult children of parents with mental illness, if any. In most cases, participants listed more than one challenge; hence frequency counts were used to tally total responses. Findings that related to the theme ‘stigma, isolation and shame’ were most frequently highlighted by participants, totalling 24 mentions. The second most cited challenge related to ‘continual worry and negative emotions’ (i.e., distress, trauma, emotional regulation), totalling 22 mentions, as noted by a participant: ‘*feeling a sense of concern or feeling worried 24/7 without reason* and *there is so many [challenges] to list.’* The third most reported challenge was best reflected under the theme: ‘issues relating to family-of-origin’. A total of 20 comments were obtained from participants that had content relating to family relationships with parents and siblings along with ongoing blame and guilt, with an adult child stating the following: ‘*in my case, both my parents had significant mental health challenges. As a result, every area of my development has been impacted negatively, such as, establishing boundaries, healthy relationships, self-care…*’.

### Program Necessity

Participants were invited to express their views on whether a support program was needed for adult children of parents with mental illness. All academics (*n* = 17) and clinicians (*n* = 12) responded ‘yes’ to the statement. Amongst adult children, 93.8% (15 out of 16) concurred with the need for further supports to continue across the lifespan, with only one participant expressing that more time and resources be dedicated to supporting young children of parents with a mental illness:

I don't feel there is a need for additional support [for adult children]. I think the support needs to always be with children. If my parents weren't so caught up in their own lives and circumstances, they might have been able to offer me tips to manage my anxiety. Or they might have had the emotional space to sit with me and talk me through my panic attacks.

### Program Delivery, Format and Duration

Participants were asked about their preferred delivery, format, and duration of a program. Adult children rated individual therapy (*n* = 13) as their most preferred format of intervention, followed by a psychoeducational package (*n* = 9) and online forums (*n* = 9). Conversely, psychoeducation packages emerged as the top-rated format, with 15 out of 17 academics and all clinicians rating it as their most preferred choice. Collectively, results from round one highlighted a diverse range of program formats that were rated favourably across expert groups. Despite ranking differences between expert groups, individual therapy, psychoeducation packages, online forum and group therapy emerged as desirable program formats. Participants were also asked about their preferred program duration. Frequently cited responses included programs that were between one to two hours per session, spanning across an average of five to eight sessions in total. Collectively, there appeared to be a preference for program brevity.

### Program Content

Participants were invited to provide content or topic areas they considered important in a program for adult children. Responses to the open-ended question were thematically analysed and summarised in Table 3. The order of themes presented in Table 3 is not presented to reflect order of priority or interest; rather, similar themes between groups were mapped together to show convergence. Given the similarity in responses between academics and clinicians, the suggested topics for both groups have been consolidated and compared to responses provided by adult children. Where similar responses were not corroborated by another expert panel group, these have been left empty to signify novelty in responses obtained.

**Table 3:** Prospective topic areas

Theme	Adult children (*n* = 16)	Academics and Clinicians (*n* = 29)
1	Mental health and self-care	–
2	Setting appropriate relational boundaries	Navigating family relationships
	Managing multiple roles	
3	Psychoeducation about parental mental illness (PMI)	Information about community and other support services to cope with PMI
4	Objectifying the illness	Education and treatment information *(mental illness or disorder specific)*
5	Establishing support networks	Learning through shared experiences
6	Emotional management	Regulation and acceptance
7	De-stigmatising PMI	Empowerment through increased knowledge about parental mental illness
		Addressing misconceptions common among children (adult children) of parents with mental illness
8	Transition to parenthood	–
9	Future planning (e.g., finances, physical health, mental health, accommodation, and day-to-day living)	–

A total of nine content areas were derived across expert groups. Most content areas (i.e., 6 out of 9) highlighted by academics and clinicians were corroborated by adult children with lived experience, suggesting a good degree of convergence and understanding about the needs of adult children. However, content such as ‘mental health and self-care’, ‘transition to parenthood’ and ‘future planning’, were emphasised by adult children but not identified by academics and clinicians in the current study. These group distinctions are important to account for in program development, to ensure it caters to the specific needs of prospective end users.

### Ways to Promote Program Participation

Table 4 summarises participant responses, in relation to factors that might promote adult children’s participation in a program. Participants acknowledged a need for a program to provide a safe space to hold personal and emotional conversations. Other factors included collaborations with health practitioners as well as hybrid delivery formats (e.g., combination of online and in-person delivery).

**Table 4:** Ways to promote program participation

Sub-themes (*extracted from responses provided by panel members*)		
Themes	Adult children (*n* = 16)	Academics/Clinicians (*n* = 29)
Provision of a safe place with peers	To share experiences with others with similar backgrounds To process one’s loss with others who have been through similar lived experiences	Non-judgemental platform Safe space that offers a sense of familiarity
Public awareness about program	Availability and awareness of programs at different levels (e.g., community centres, hospitals, clinics) Advocates to champion mental illness and anti-stigma campaigns	–
Collaboration with practitioners	Doctor/mental health practitioner participation in raising awareness about available programs or support avenues Coordination with community service providers (i.e., GP, maternal child and health nurse)	Provision of subsidies especially for those living in rural areas (i.e., making interventions accessible)
Flexibility in program modality	Not an excessive time commitment Online learning Flexibility in rate of participation (adult children may be hard pressed for time and energy) Option of both online and face-to-face options Able to access content, as and when needed (on-demand service)	Have a range of mediums to facilitate access Scheduling it at an opportune time (e.g., evenings and on a monthly basis)
Program conducted by individual with lived experience	Content designed and delivered by lived experience experts Non-judgemental and understanding facilitators	Relevance and first-hand awareness about problems and challenges faced by adult children of parents with mental illness A source of recognition and validation
Opportunities to build support networks	Developing friendships, connections with moderators and other participants An avenue to receive peer support and professional inputs	Involvement of wider community Cultivating a sense of shared experience A sense of hope could be cultivated by seeing others in similar positions who have successfully adapted and worked at their problems Practitioners can aid in fostering some leaders or champions within support groups to be advocates for other adult children

### Program Facilitator Qualifications

Across all groups, participants agreed that a facilitator should be present regardless of whether the program was conducted face-to-face or online. Approximately half the responses (i.e., 21 out of 45 panellists) cited the need for a facilitator to have both lived experience and mental health qualifications. Notably, although more academics (n = 7) rated lived experience alone to be an important attribute for a moderator to have, this was not endorsed as strongly by clinicians (n = 1) and adult children (n = 1). For the latter groups, the presence of a trained mental health professional was preferred more than a facilitator with lived experience alone.

Panellists rated the following responses as important skillsets for facilitators to possess: (i) relevant clinical knowledge (n = 28), (ii) counselling and therapeutic skills (n = 26), and (iii) group facilitation experience (n = 22). Participants viewed group facilitation skills as a necessary precursor in being able to effectively manage and regulate potentially sensitive conversations. These results pertaining to qualification and skillsets of a facilitator were the same regardless of whether it was a face-to-face or online.

### Preference for Online Forums

Panellists’ opinions were also solicited about the level of interest in an online forum to support adult children of parents with mental illness. Most clinicians (83.3%) and academics (100%) responded ‘yes’ to the provision of online forums. Amongst adult children, 87.5% responded positively the use of online forums as a source of support. Amongst the minority of participants who responded ‘no’, one participant with lived experience stated the following: *“we need public awareness around this issue to support kids and young people who are currently experiencing this [parental mental illness]. A closed forum does nothing for this [raising public awareness].”* Clinicians, who responded ‘no’, expressed a lack of familiarity with online forums, or considered such platforms only as a site to house supplementary material.

### Online Forums and Anonymity

In relation to anonymity on online forums, 91.6% of clinicians, 88.2% of academics and 87.5% of adult children agreed to keeping identities anonymous. However, the following excerpt highlights divergence amongst those who were unconvinced about anonymity on online forums: *“people need to speak up and start using their real names to eliminate trolls”* and *“people should have the option but if a forum demands anonymity, it suggests that to be identified would be harmful/shameful and therefore plays into stigma”.*

## Results: Round Two

Round two primarily included ‘rating’ or ‘ranking’ questions, to allow for responses to be analysed quantitatively (Thangaratinam & Redman, 2005). Each question was followed up with a free response text box that allowed participants the option to provide qualitative comments, if needed.

### Program Format

Across expert panel groups, individual therapy was consistently ranked at first place for preferred program format (Table 5). Although academics and clinicians were of the view that psychoeducation of various modalities would be a viable program format, adult children did not agree. Instead, adult children rated public awareness campaigns and group therapy as second and third choices respectively. These findings suggest that adult children, at least in the current study, assign more importance on efforts that promote education and awareness about their experiences as an adult child compared to general psychoeducation about parental mental illness.

**Table 5:** Top three panel rankings for program formats

Expert panel group	Top 3 preferred program format		
	Rank 1	Rank 2	Rank 3
Academics	Individual therapy ***and*** Public awareness campaigns	Psychoeducational materials (online)	Peer networking sessions
Clinicians	Individual therapy ***and*** Peer networking sessions	Group psychoeducation	Support groups (flexi drop-in sessions)
Adult children	Individual therapy	Public awareness campaigns	Group therapy (in-person)
Combined Panel^a^	Individual therapy	Public awareness campaigns	Peer networking sessions

^a^Aggregated rank across expert panel groups

### Program Duration and Frequency

There was a unanimous ranking that a prospective program should ideally be between five to eight sessions in duration. In terms of program frequency, although academics ranked weekly sessions first, clinicians and the lived experience group members preferred fortnightly or monthly programs. Collectively, these ranks suggest that adult children may prefer a program that is relatively spaced apart. This need was further corroborated by a comment obtained from an adult child: “*not a significant time commitment due to full time work/study commitments and family of origin/family of choice responsibilities.”*

### Facilitator Qualifications

Panellists were asked to rank the facilitator qualifications they felt was most important. As outlined in Table 6, all groups regarded an individual with mental health training combined with lived experience as an adult child of a parent with mental illness to be the most important attribute. Second and third choice ranks, however, differed amongst expert panel groups. Although academics and clinicians ranked group facilitations skills as second, those with lived experience ranked mental health training as the second most important attribute. The third rank was awarded to group facilitation skills. The difference in second and third ranks suggests that for people with lived experience, a facilitator with mental health training was important. Although it was preferred that the facilitator would have both mental health training and lived experience, when presented with either of those options independently, adult children prioritised a facilitator with mental health training over someone with lived experience only.

**Table 6:** Top panel ranking for program facilitator qualifications

Expert panel group	Top 3 preferred facilitator qualifications		
	Rank 1	Rank 2	Rank 3
Academics	Mental health training with lived experience	Group facilitation skills	Professional training in mental health only
Clinicians	Mental health training with lived experience	Group facilitation skills	Lived experience only
Adult children	Mental health training with lived experience	Professional training in mental health only	Group facilitation skills

### Content Areas of Interest

Table 7 highlights content areas that were identified as important across expert panel groups. A total of ten topic areas met consensus criteria (i.e., mean rating of 4.0 or greater, SD 0.85 or less). ‘Mental health and self-care’ was the only topic that all panellists agreed as important to include. Other topics, such as psychoeducation and de-stigmatisation of parental mental illness were rated highly by academics and clinicians but not by adult children. Importantly, five topics were endorsed by adult children, including: (i) mental health and self-care, (ii) managing multiple roles and navigating family relationships, (iii) emotional management, regulation and acceptance, (iv) setting relational boundaries and (v) transition to parenthood.

**Table 7:** Top panel ratings for content/topics of interest

Content area	Mean (SD)		
	Academics	Clinicians	Adult children
Mental Health and self-care^a^	4.14 (0.69)	4.63 (0.52)	4.78 (0.44)
Psychoeducation about PMI	4.43 (0.53)	4.38 (0.74)	–
De-stigmatization of PMI	4.43 (0.53)	4.13 (0.35)	–
Managing multiple roles and navigating family relationships^a^	4.00 (0.82)	–	4.56 (0.73)
Emotional management, regulation, and acceptance^a^	–	4.35 (0.46)	4.22 (0.67)
Setting relational boundaries^a^	–	4.00 (0.76)	4.44 (0.53)
Addressing misconceptions and stigma about mental illness	4.57 (0.53)	–	–
Information about community/other support services	4.29 (0.49)	–	–
Establishing peer support networks	–	4.38 (0.52)	–
Transition to parenthood^a^	–	4.13 (0.64)	4.00, (0.85)

^a^Topics that were endorsed by adult children

### Ways to Promote Program Participation

As outlined in Table 8, individuals with lived experience expressed a need for programs to be promoted through grassroots channels, such as community centres, medical clinics, and hospitals. Additionally, participants with lived experience reported that collaborations with various mental health practitioners and/or organisations was another means to promote awareness about support programs and consequently enhance participatory rates. For academics and clinicians, the provisions of support programs to rural communities, as well as access to a non-judgmental, safe place where adult children could learn from shared experiences, were seen as factors that could increase participation rates.

**Table 8:** Ways to promote program participation

Content area	Mean (SD)		
	Academics	Clinicians	Adult children
Collaborations with health practitioners and/or organisations to increase awareness about support provisions/programs	4.29 (0.49)	4.12 (0.35)	4.00 (0.71)
Community-level programs	4.29 (0.49)	–	4.22 (0.67)
Option of a flexible program and is not an excessive time commitment	4.43 (0.79)	4.13 (0.64)	–
Provision of subsidies to individuals living in rural areas	4.00 (0.82)	4.13 (0.64)	–
A safe place where adult children can gather and build on shared experiences	4.29 (0.76)	4.25 (0.71)	–

## Discussion

Some adult children of parents with mental illness experience ongoing challenges at different life stages, including marriage, and parenthood (Murphy et al., 2011). To address the lack of support for this group, three expert panel groups (i.e., academics, clinicians and adult children) were convened to obtain a multi-stakeholder perspective on the types of support that could be provided to adult children of parents with a mental illness. The inclusion of insights from individuals with lived experience, specifically when dealing with complex issues, such as parental mental illness is valuable when developing programs and services (Daya et al., 2020).

### Program Content

A total of ten content areas met the consensus criteria across expert panel groups. Out of the ten areas, five topics were endorsed by individuals with lived experience. In a broad sense, the five content areas could be classified into intrapersonal and interpersonal relationship issues. Within the intrapersonal domain, a central tenet that emerged in both rounds of the Delphi study related to self-care. Self-care is an important area for adult children, who may be physically and emotionally burdened from caregiving responsibilities towards their parent with mental illness as well as their own children. These findings corroborate with those obtained by Smith et al. (2018), who explored the needs of individuals who cared for children with life-threatening illnesses. Smith et al. (2018) reported a desire amongst carers to schedule personal time for them, to give themselves permission to take a break and to step away from caregiving duties. Similarly, a meta-analysis by Jeon et al. (2005) found that family caregivers of individuals with severe mental illness required respite breaks to maintain their own health and wellbeing. Drawing parallels, the emphasis placed on mental health and self-care by participants in this study could be suggestive of a desire amongst adult children to attain some personal time and respite from having to manage and balance multiple familial and caregiving responsibilities.

Relatedly, managing family relationships and multiple responsibilities included sub-topics such as supporting adult siblings in caring for a mentally ill parent, managing multiple caring roles (caring for parent and own child) and decision-making about caregiving arrangements for the parent with mental illness. Furthermore, it is likely that adult children are not only caring for a parent with a mental illness, but an *elderly parent* with a mental illness. Previous literature also suggests that an older person with mental illness is vulnerable to other issues in conjunction to their mental illness, such as age-associated risk factors, including cognitive deficits and/or physical ailments (Bartels, 2004). Collectively, these factors could present with unique and specific challenges that adult children might experience and require ongoing supports with.

‘Setting relational boundaries’ was another topic endorsed by adult children, which embodied sub-topics such as, learning to set boundaries in the parent–child relationship, and establishing limits to what they would accept from their parent’s behaviour towards themselves and their family of procreation. Adult children also expressed wanting information on what healthy relationships with their parent might look like and practical steps they could put in practice to achieve that. Other studies have found that young adults (aged 18–25) whose parents have a mental illness also expressed similar sentiments (Matar et al., 2018), suggesting this as a need across the trajectory of adulthood.

In their narrative inquiry with adult children, Murphy et al. (2016) suggested that participants experienced “loss and becoming lost as a person” (p. 672) and felt disconnected from reality and to some extent out of touch with their own emotions. Others have found that the development of autonomy and independence is accompanied by feelings of guilt and ambivalence about caring for their parent (Metz & Jungbauer, 2019). The findings here suggest that a lost sense of self, and by extension, a desire to form an identity outside of their parent’s mental illness remains a need for these individuals. For adult children, skills to establish healthy boundaries may allow for cordial and respectful relationships between themselves and their parents. Such relationships may circumvent the conflicted/ambivalent feelings that may arise from having a life outside of their parent’s mental illness.

Another content area requested by adult children and supported by clinicians related to ‘transition to parenthood’. Foster (2010) noted that adult children’s experiences with parental mental illness had an ongoing impact on their interpersonal connections with spouses and children. Although adult children experienced several interpersonal challenges as parents, adult children also attached a large part of their identity on being parents (Patrick et al., 2019). Despite some degree of interpersonal difficulties (Foster, 2010) and parenting self-doubt (Murphy et al., 2018), some adult children ultimately view parenthood both as a protective factor to their own mental health and wellbeing and regarded it as an opportunity to start afresh with their children. Thus, a program that includes specific strategies on parenting skills and serves as a platform for adult children to connect with others of similar lived experience could mitigate and normalise parental anxieties, worries and challenges. This finding reiterates the importance of supporting family members throughout the lifespan, as a new developmental stage may present individuals with difficult and different challenges over time (Metz & Jungbauer, 2019).

### Program Format

Panellists agreed that individual therapy and a variation of group therapy or group psychoeducation would be of benefit to adult children. Adult children ranked individual therapy as their most preferred format, which could be useful in working through potentially sensitive or private issues. Another program format that emerged across panellists was for group-based programs, which may be suggestive of a desire to connect with others of similar familial backgrounds. Previous studies found that children were more likely to talk about their parent’s illness amongst peers in a peer-led program, as it felt safe and ‘normal’ (Grové et al., 2016). Given the preference for individual and group-based programs, future initiatives could explore a hybrid approach where some topics that are potentially sensitive and varied could be addressed in individual sessions. Conversely, topics such as ‘managing family relationships and multiple responsibilities’ or ‘setting relational boundaries’ could be topics addressed in group-led programs as this could offer adult children a platform to also hear and learn from the lived experiences of others. Furthermore, extant literature has documented other benefits of peer support, including expansion of social networks, feelings of empowerment and self-determination and stigma reduction through empathetic understanding and exchange of personal experiences (Chapin et al., 2013).

For most adult children, public awareness programs, especially those with a focus on how mental illness can impact a family unit, was seen as a necessary step in reducing stigma and encouraging families to seek help. Children and adult children of parents with mental illness are often recipients of “associative stigma” (Koschade & Lynd-Stevenson, 2011), which is inflicted on an individual due to his or her association to another stigmatised individual (Goffman, 1963). The current findings accentuate Goffman’s (1963) stance that associative stigma is felt more strongly if the relationship between two individuals is closer in proximity (e.g., parent and child) as opposed to one that is distant (e.g., extended family member). Relatedly, some adult children reported that having an anonymised forum might suggest that their lived experience needed to be kept a “secret”, which could potentially be stigmatising.

### Program Duration and Facilitator Qualifications

Program brevity and flexibility were key features that adult children identified. Some adult children wanted flexibility in the modules they attended, so they could choose topics they had a personal interest or need in. Additionally, across all expert panel groups, consensus was for a facilitator to have both mental health training and lived experience of parental mental illness. Thus, it may prove beneficial for a prospective program to consider facilitators with professional training and lived experience, or the use of multiple facilitators, each with a specific area of specialization to offer different perspectives and experiences.

### Study Strengths and Limitations

The current study provides preliminary suggestions on content and modality for a support program targeted towards adult children. Importantly, a key strength of this study is that adult children were invited to provide their opinions regarding what they believe to be important to include in a prospective support program. Several limitations should be noted. Firstly, it is possible that panel members who personally thought such support services or programs to be important were those who ultimately offered their views through the Delphi study. Secondly, the decision to conduct a two-round Delphi study is a limitation of this study. A third round of responses where panellists compared their ratings to the aggregated rating/ranking across panel members would have allowed the opportunity for each expert member to reflect and amend their initial ratings. However, given attrition rate from the first to second round, and research that indicates “repeated rounds may lead to fatigue by respondents and increased attrition” (Thangaratinam & Redman, 2005, p.122), the choice was made to cease the Delphi study following round two. Future studies could replicate the current Delphi study by including panel members across socioeconomic strata, cultural, linguistic, and other diverse groups.

## Conclusion

The Delphi findings highlighted topic area and program delivery formats that should be considered when designing support programs to address the specific needs of adult children of parents with a mental illness. The results of this study provide a foundation to inform the development of modules and initiatives that could be pilot tested with a small group of adult children.
